# Differentiation of Apical and Basal Dendrites in Pyramidal Cells and Granule Cells in Dissociated Hippocampal Cultures

**DOI:** 10.1371/journal.pone.0118482

**Published:** 2015-02-23

**Authors:** You Kure Wu, Kazuto Fujishima, Mineko Kengaku

**Affiliations:** 1 Graduate School of Biostudies, Kyoto University, Kyoto, Japan; 2 Institute for Integrated Cell-Material Sciences (WPI-iCeMS), Kyoto University, Kyoto, Japan; Osaka University Graduate School of Medicine, JAPAN

## Abstract

Hippocampal pyramidal cells and dentate granule cells develop morphologically distinct dendritic arbors, yet also share some common features. Both cell types form a long apical dendrite which extends from the apex of the cell soma, while short basal dendrites are developed only in pyramidal cells. Using quantitative morphometric analyses of mouse hippocampal cultures, we evaluated the differences in dendritic arborization patterns between pyramidal and granule cells. Furthermore, we observed and described the final apical dendrite determination during dendritic polarization by time-lapse imaging. Pyramidal and granule cells in culture exhibited similar dendritic patterns with a single principal dendrite and several minor dendrites so that the cell types were not readily distinguished by appearance. While basal dendrites in granule cells are normally degraded by adulthood *in vivo*, cultured granule cells retained their minor dendrites. Asymmetric growth of a single principal dendrite harboring the Golgi was observed in both cell types soon after the onset of dendritic growth. Time-lapse imaging revealed that up until the second week in culture, final principal dendrite designation was not stabilized, but was frequently replaced by other minor dendrites. Before dendritic polarity was stabilized, the Golgi moved dynamically within the soma and was repeatedly repositioned at newly emerging principal dendrites. Our results suggest that polarized growth of the apical dendrite is regulated by cell intrinsic programs, while regression of basal dendrites requires cue(s) from the extracellular environment in the dentate gyrus. The apical dendrite designation is determined from among multiple growing dendrites of young developing neurons.

## Introduction

Neurons of the central nervous system exhibit enormously diverse dendritic arbor architecture, which determines both the number and type of synaptic inputs received, and hence critically affect neuronal connectivity. Pyramidal cells in the Ammon’s horn (cornu ammonis, or CA) and granule cells in the dentate gyrus (DG) are the two principal neuronal types in the hippocampal formation, exhibiting distinct dendritic arbor structures. Pyramidal cells give rise to a long, thick apical dendrite and several minor basal dendrites that emerge from the apex and base of the teardrop-shaped soma, respectively. The apical and basal dendrites are oriented in opposite directions and occupy different layers, with apical dendrites extending toward the hippocampal fissure through the stratum radiatum and stratum lacunosum-moleculare, and basal dendrites extending in the opposite direction through the stratum oriens. Thus, the dendritic architecture of pyramidal cells take a biconical shape and are able to receive synaptic inputs from different afferent sources. In contrast to pyramidal cells, dentate granule cells have a monoconical arbor of apical dendrites with all branches directed toward the superficial region of the molecular layer. Immature granule cells produce transient dendrites from the basal portion of the soma, which are then retracted by adulthood, except for in a small population of granule cells in primates [1–3].

Apical and basal dendrites differ in various properties, including size, geometry, electrical conduction, and responsiveness to neurotrophic factors or guidance molecules [4–6]. It has been shown that extension and branching of apical and basal dendrites are differentially regulated by the secreted guidance molecule, semaphorin 3A [7–12]. Accumulating evidence has also implicated preferential localization of the Golgi apparatus to the base of the apical dendrite during establishment of dendritic polarity [13, 14]. Other extrinsic and intrinsic cues involved in the control of dendritic polarity have emerged, but the precise mechanism of how pyramidal and granule cells initiate, develop and maintain apical and basal dendrites remain elusive [15].

Pyramidal cells in primary culture establish dendritic polarity with a single thick, elongated dendrite and several short dendrites similar to pyramidal cells *in vivo* [16]. Primary cultures of dentate granule cells have also been established, but less is known about the specification of dendrites in cultured granule cells [17]. Here, using long-term time-lapse imaging and quantitative morphometric analysis in primary cultures, we comparatively analyzed the dynamics of dendritic differentiation in pyramidal and dentate granule cells.

## Materials and Methods

### Animals

ICR mice for primary hippocampal culture were obtained from Japan SLC, Inc.. All procedures involving mice were performed in strict accordance with the institutional guidelines and approved by the Animal Experimentation Committee of Kyoto University (Permit Number: icems-2-21). All surgery was conducted under anesthesia using isoflurane for adults, or deep hypothermia for pups, and all efforts were made to minimize suffering.

### Primary neuron culture

Primary cultures of hippocampal neurons were prepared as previously described with a few modifications [18, 19]. In brief, hippocampi were dissected from mice aged from E17 to P4. Hippocampal neurons were dissociated by using SUMITOMO Nerve-Cell Culture System (Sumitomo Bakelite) and plated on coverslips or glass-based dishes coated with poly-D-lysine at a density of 1.0–2.0 x 10^5^ cells/cm^2^ in MEM supplemented with 10% horse serum (Gibco), 0.6% D-glucose, 1 mM sodium pyruvate and 1% penicillin-streptomycin. Three hours after plating, media was replaced by Neurobasal medium (Gibco) supplemented with B-27 supplement (Gibco), 0.5 mM L-glutamine and 1% penicillin-streptomycin. All neurons were maintained at 37°C in 5% CO_2_. Neurons were transfected with Lipofectamine 2000 (Invitrogen) according to the manufacturer’s instructions. pCAGGS-GRASP65-GFP was transfected at DIV 4. Other constructs were transfected at DIV 2–4.

### DNA constructs and antibodies

pCA-EGFP and pAAV-CAG-TdTomato were previously described [20, 21]. pAcGFP1-Golgi was obtained from Clontech. For construction of pCAGGS-GRASP65-GFP, cDNA encoding GRASP65 was cloned by PCR from a cDNA library. The PCR product was subcloned into pEGFP-N1 vector (Clontech), and the EGFP-fused GRASP65 was subcloned into pCAGGS vector. Antibodies used for immunostaining were as follows: rabbit anti-Math2, anti-Prox1 and anti-Synapsin I, chick anti-NeuN (Abcam); mouse anti-GM130 (BD Biosciences); rabbit anti-Calbindin, anti-GFAP and mouse anti-GAD67 (Chemicon); mouse anti-PSD95 (Funakoshi); mouse anti-Prox1 (Millipore); goat anti-Ankyrin G (Santa Cruz); rabbit anti-Calretinin (Swant); Alexa405-, Alexa488-, Alexa568-, Alexa633- or Alexa647-conjugated anti-chick, anti-goat, anti-mouse or anti-rabbit IgG (Molecular Probes).

### Immunofluorescence and morphological analysis

Cells were fixed with 4% paraformaldehyde in PBS and permeabilized with 0.3% Triton X-100 in PBS. Cells were then blocked with blocking solution (2% skim milk, 0.1% Tween20 in PBS) and incubated with the primary antibodies at 4°C overnight. After washing with PBS, cells were incubated with secondary antibodies at 4°C overnight and stained with DAPI at room temperature for 10 minutes. Morphologies of immunostained cells were analyzed by a laser-scanning confocal microscope FV1000 (Olympus) with a 20× dry objective (N.A. 0.75, Olympus), 40× dry objective (N.A. 0.95) and a 100× oil-immersion objective (N.A. 1.4). Detailed methods for immunostaining and confocal analyses of labeled neurons were described previously [18].

Axon/dendrite specification was identified by immunostaining with anti-Ankyrin G and the morphological criteria described previously [22, 23]. Cells that were clearly free from other transfected cells were selected and analyzed by virtue of soluble GFP or TdTomato fill. Dendrites were traced with the aid of Neurolucida software (MBF Bioscience) and processed for quantitative analysis using Neurolucida Explore (MBF Bioscience) and ImageJ (NIH). The principal dendrite was defined as the longest dendrite of a neuron. To analyze the localization of the Golgi apparatus, the nucleus center of mass was set as a polar coordinate origin, and the direction to the base of the principal dendrite was defined as *φ* = 0. A neuron was separated into three regions by *φ* interval: (–π/4, π/4] as the apical region, (π/4, 3π/4] and (-3π/4, -π/4] as the lateral region, and the remaining interval as the basal region. The area occupied with the GM130 signal in each region relative to the whole GM130 area in the soma was calculated. All data are expressed as mean ± SEM.

### Time-lapse imaging

For time-lapse imaging, neurons from P0 mice were plated on a glass-based dish and transfected with indicated plasmids encoding fluorescent markers. Labeled cells were observed every 15 or 30 minutes with a spinning-disc confocal microscope CV1000 (Yokogawa) through a 20× dry objective (N.A. 0.75, Olympus) or a 40× oil-immersion objective (N.A. 1.3, Olympus). All neurons were imaged at 37°C with constant gas flow with 5% CO_2_ and 95% air.

## Results

### Morphologies of neurons in the primary culture of the mouse hippocampal formation

The primary culture of hippocampal neurons was prepared from the hippocampal formation of neonatal mice at postnatal day 0 (P0). Cultures were fixed after 2 days *in vitro* (DIV 2) for immunofluorescence using cell-type specific markers, which include pyramidal cell marker Math2 [24, 25], granule cell marker Prox1 [26, 27], interneuron marker GAD67 [28] and GFAP for glial cells [29]. The expression of Math2 and Prox1 was mutually exclusive (S1 Fig.). Approximately 25% of the cultured cells were Math2-positive pyramidal cells and another 25% were Prox1-positive granule cells. GAD67-positive interneurons accounted for less than 10% of cells in the culture. GFAP-positive glia constituted about 50% of cells in the culture.

**Fig 1:**
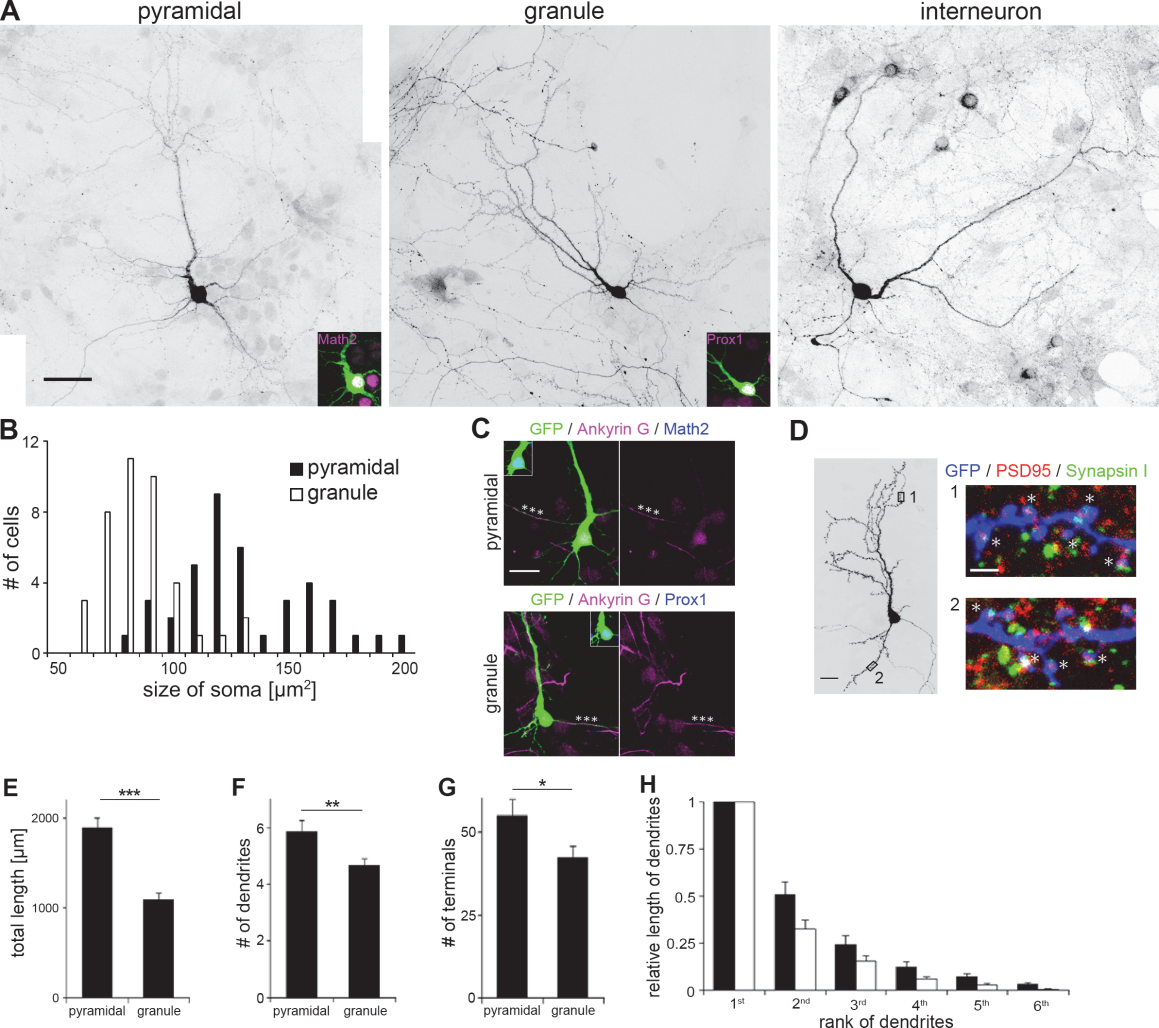
The morphology of pyramidal and granule cells at DIV 13 in dissociated culture. (A) Typical morphology of pyramidal cells, granule cells and interneurons. The cell type was characterized by immunostaining with Math2, Prox1 (magenta) and GAD67, respectively. Pyramidal and granule cells were visualized by GFP transfection. Interneurons were visualized by immunostaining with GAD67. Scale bar: 40 μm. (B) Histogram distribution of somal size in pyramidal and granule cells of DIV 10–13. n = 40 for each cell type. (C) Axon/dendrite specification as demarcated by Ankyrin G expression. Cells were immunostained with Ankyrin G (magenta) and Math2 or Prox1 (blue) at DIV 7. Asterisks indicate axon. Scale bar: 20 μm. (D) Expression of synaptic markers at DIV 16. Boxed regions in the left panel are shown in the right panels. PSD95 (red) was localized in protrusions in both principal (upper) and minor (lower) dendrites. Some PSD95 signals in dendritic protrusions (asterisks) were apposed to Synapsin I signals (green). Dendrites were visualized by GFP expression. Scale bar: 20 μm (left), 2 μm (right). (E-G) Quantification of total dendritic length (E), the number of primary dendrites (F) and branch terminals (G). n = 20. Student's t-test: *P<0.05, **P<0.03, ***P<0.001. (H) Ratio of length of each dendrite arbor to the longest dendrite. All dendrite arbors in a neuron were ranked from the longest to the shortest. n = 20.

By the second week *in vitro*, pyramidal cells have a large soma with an average area of 154.5 ± 38.6 μm^2^ (*n* = 40) while granule cells have a smaller soma of 87.5 ± 19.1 μm^2^ in area (*n* = 40) (Fig. 1A and 1B). Pyramidal and granule cells exhibited similar morphology with a single long, thick primary dendrite (referred to as principal dendrite hereafter) and several shorter dendrites. We confirmed that these dendrites were distinct from the axon expressing an axonal marker Ankyrin G (Fig. 1C). Both principal and minor dendrites bore numerous protrusions expressing a post-synaptic marker PSD95 (Fig. 1D). These dendritic protrusions were frequently juxtaposed with punctate signals of a pre-synaptic marker Synapsin I, suggesting that the neurons in the dissociated culture developed mature dendrites with functional synapses. The overall size of the dendritic arbor was larger in pyramidal cells than granule cells, as indicated by the total length of dendritic branches and the number of primary dendrites and branch terminals (Fig. 1E-1G). The asymmetry of dendritic arbors was more prominent in granule cells such that the longest dendrite contributed 67% of the total dendritic length in granule cells, in contrast to 53% in pyramidal cells (Fig. 1H).

In contrast to pyramidal and granule cells, GAD67-positive interneurons extended several equivalent dendrites with no clear dendritic polarity as previously reported (Fig. 1A) [16].

### Differentiation of pyramidal and granule cells in culture

**Fig 2:**
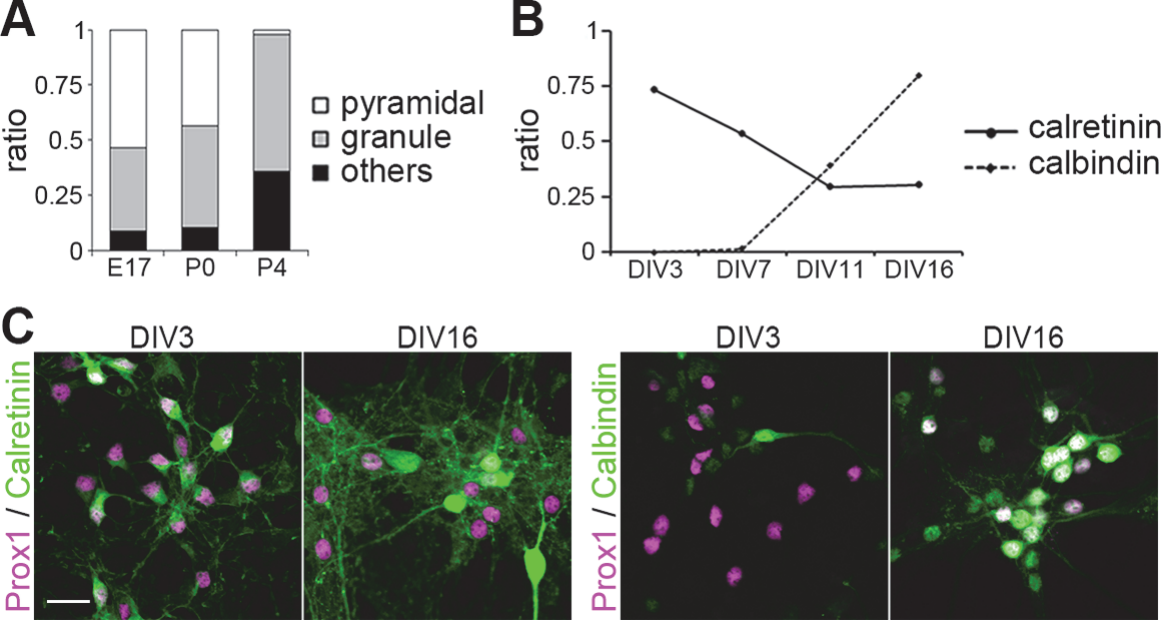
Population of pyramidal and granule cells in dissociated culture. (A) Proportion of neuronal species in cultures from different aged pups. Whole neurons, pyramidal cells and granule cells were identified as NeuN-, Math2- and Prox1-positive cells, respectively. n = 722 (E17), 508 (P0), 634 (P4). (B) Maturation of granule cells. Each point shows the ratio of each marker-positive cells to Prox1-positive cells. n (DIV 3, DIV 7, DIV 11, DIV 16) = (371, 202, 258, 95) for calretinin, (395, 186, 208, 123) for calbindin. (C) Maturation marker expression of granule cells. Cells were immunostained with calretinin or calbindin (green) and Prox1 (magenta). Scale bar: 30 μm.

Dentate granule cells and hippocampal pyramidal cells originate from adjacent but distinct regions in the neuroepithelium in the dorsal telencephalon, also called the medial pallium, with the primordium of the DG lying in the medial-most region, followed by the CA fields and the subicular cortex [30–32]. It has been shown that differentiation of pyramidal and granule cells starts almost simultaneously at embryonic day 10 (E10) in the mouse hippocampal formation [33]. The generation of pyramidal cells completes at around E18, while granule cell production continues until adult stages. We thus questioned if the ratio of pyramidal and granule cells changed in cultures prepared from hippocampi at different ages. We found that the proportion of pyramidal cells was higher in cultures prepared from younger mice (Fig. 2A). In cultures prepared from E17 mice, Math2-positive pyramidal cells accounted for 53% of all neurons, while 38% were Prox1-positive granule cells. Pyramidal cells sharply decreased in cultures prepared from postnatal brain and were not observed in cultures prepared from P4 mice. In contrast, the proportion of Prox1-positive granule cells slightly increased with age, reaching 62% in cultures from P4 mice.

We next examined neuronal maturation in culture. It has previously been shown that immature granule cells express calretinin, while mature granule cells express calbindin and sequentially downregulate calretinin [34]. We analyzed the expression of these differentiation markers together with Prox1 in the primary hippocampal culture prepared from P0 mice. At DIV 3, the large majority of Prox1-positive granule cells expressed calretinin (Fig. 2B and 2C). Although calretinin expression gradually decreased in older culture, expression was still detectable by DIV 16 in 31% of Prox1-positive cells, unlike in mature granule cells *in vivo*. Calbindin-positive granule cells were first observed by DIV 7 and rapidly increased with age in culture, reaching 80% by DIV 16. Thus, developing granule cells in the primary hippocampal culture undergo time-dependent changes in gene expression similar to those observed *in vivo*.

**Fig 3:**
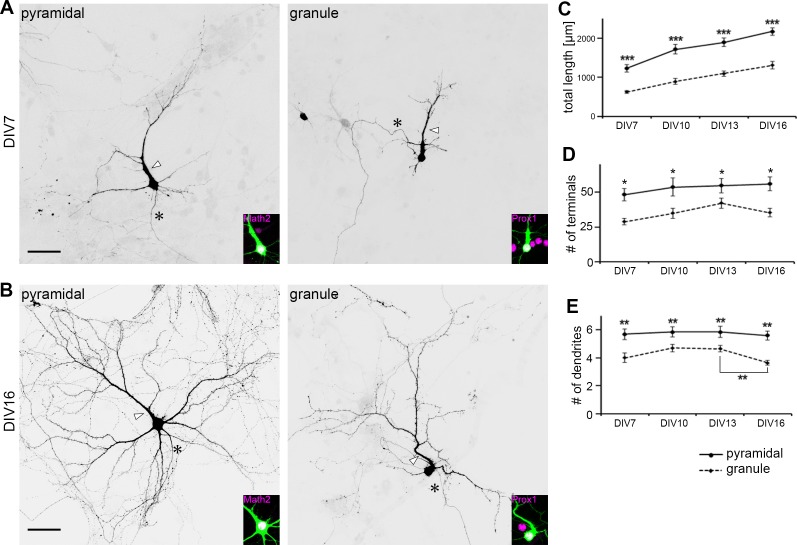
Morphology change in pyramidal and granule cells. (A-B) Typical morphology of pyramidal and granule cells at DIV 7 (A) and DIV 16 (B) of cultures prepared from P0 mice. Cells were visualized by GFP transfection. Cell types were identified by Math2 and Prox1 immunostaining (magenta). The principal dendrite and axon were indicated by white arrowhead and asterisk, respectively. Scale bar: 40 μm. (C-E) Quantification of total dendritic length (C), the number of branch terminals (D) and primary dendrites (E) during dendrite development. n = 20. Student's t-test: *P<0.05, **P<0.03, ***P<0.001.

We next compared dendritic development in pyramidal and granule cells in the primary culture prepared from P0 mice. Except for the apparent difference in size, overall branch pattern and morphology were similar in pyramidal and granule cells at DIV 7 (Fig. 3A). Both cell types had a single principal dendrite with numerous oblique branches and multiple minor dendrites that were less branched. Granule cells looked like miniatures of pyramidal cells at this earlier stage. Difference in branch patterns became more prominent in older culture by DIV 16 (Fig. 3B). Pyramidal cells had an elaborately branched principal dendrite and several minor dendrites with increased length and complexity. In contrast, granule cells at DIV 16 had a well-developed principal dendrite, while other minor dendrites remained short and bore only few branches.

For quantitative comparison of dendritic development, the number and length of all branches in Math2-positive cells and Prox1-positive cells were measured from DIV 7 to 16. The total dendritic length gradually increased in parallel, with pyramidal cell dendrites being longer than granule cell dendrites by approximately 2-fold at all stages examined (Fig. 3C). The number of branch terminals was also consistently greater in pyramidal cells, with a slight increase in both pyramidal and granule cells from DIV 7 to DIV 16 (Fig. 3D). Pyramidal cells had on average 5 or more primary dendrites at all stages examined (Fig. 3E). In contrast, granule cells had an average of 4 or 5 primary dendrites up until DIV 13, which decreased to 3 or 4 primary dendrites by DIV 16. Dendritic polarity of granule cells became prominent by this stage in culture.

### Dendrite growth dynamics in pyramidal and granule cells

**Fig 4:**
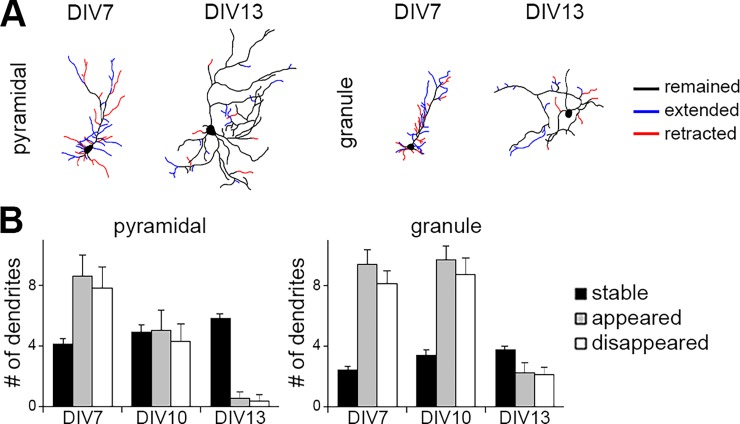
Dynamics of dendrite development. (A) Typical morphology change in pyramidal and granule cells cultured from P0 mice. Blue lines indicate newly extended dendrites, red lines indicate retracted dendrites within a 12 hr period of observation. (B) The number of dendrites during dendritic development. Each bar indicates the number of stable, appeared or disappeared dendrites within a 12 hr period of observation in 30 min-interval movies. n = 10 (DIV 7, 10), 16 (DIV 13).

Using time-lapse imaging of developing neurons in culture, we next observed the dynamic structural plasticity of dendritic arbors in pyramidal and granule cells at various stages. The primary cultures were prepared from P0 mice. At DIV 7, both pyramidal and granule cells underwent extensions and retractions of dendrites (Fig. 4A and 4B, S1 Movie). Formation and elimination of minor dendrites were frequently observed at this stage. We occasionally observed retraction of principal dendrites and substitution by other growing dendrites. Dendrites of pyramidal cells became less dynamic by around DIV 10. Primary dendrites including the principal dendrite and other minor dendrites appeared to be fixed, while dendritic tips and higher ordered branches continued to grow with repeated extension and retraction. These were mostly stabilized by DIV 13 (S2 Movie). In contrast, granule cell dendrites remained highly dynamic at DIV 10. Principal dendrites appeared to be determined, while higher ordered branches and minor primary dendrites continued extension and retraction until DIV 13 (Fig. 4A and 4B, S2 Movie). Thus, granule cell dendrites maintained structural plasticity until later stages compared to pyramidal cell dendrites.

### Specification of the apical dendrite in pyramidal cells and granule cells

Previous studies using rat neurons have indicated that dendrites are not polarized but equivalent in length until DIV 8 [13, 16]. In contrast, in our culture prepared from P0 mice, dendritic polarity was already prominent by DIV 7 in both pyramidal and granule cells. We thus questioned when dendritic polarity was established in cultured mouse hippocampal neurons by using time-lapse imaging.

**Fig 5:**
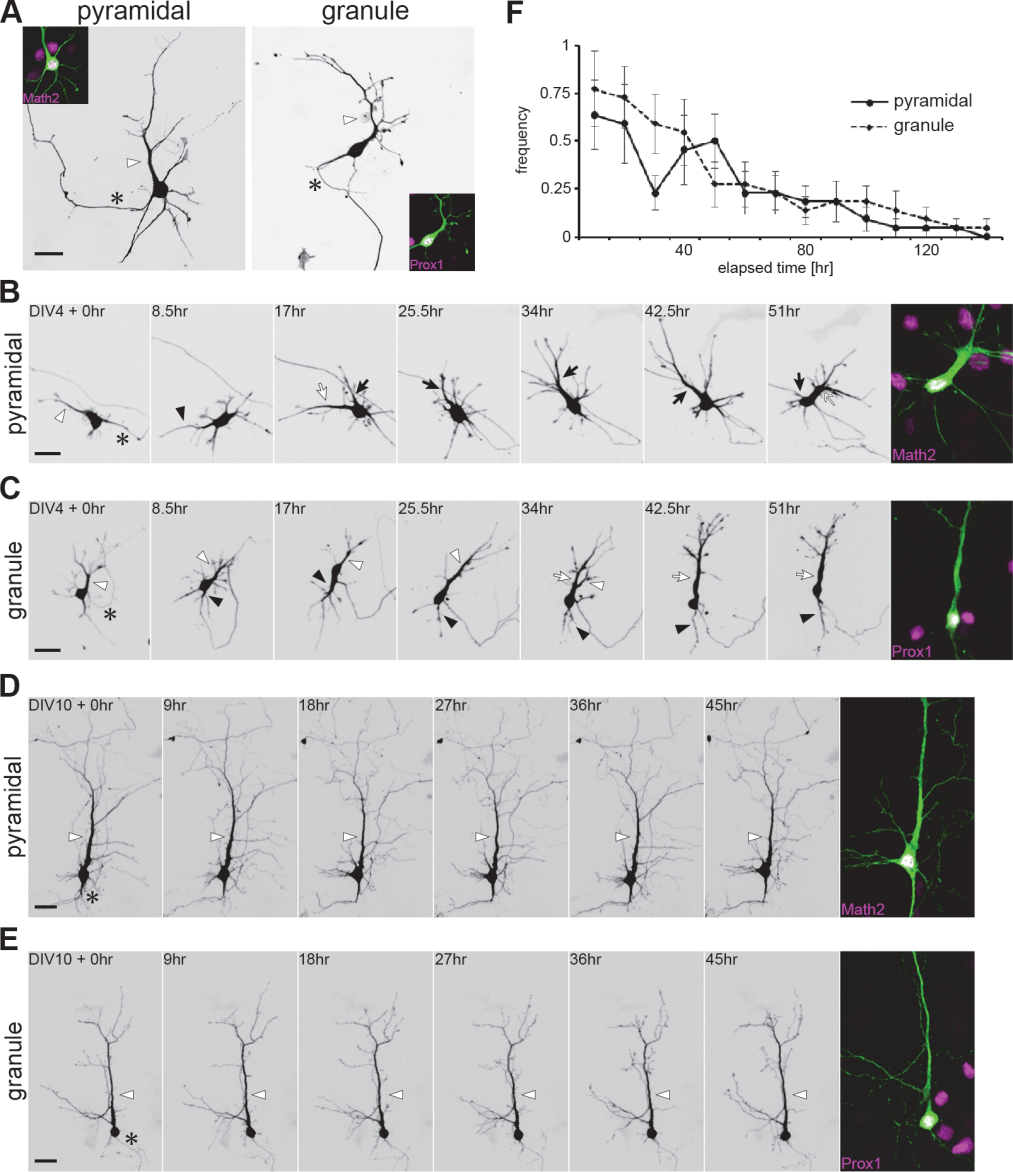
The principal dendrite determination. (A) Typical morphology of the pyramidal and granule cell at DIV 4. Cells were isolated from P0 hippocampi and visualized by GFP transfection. Cell types were identified by Math2 and Prox1 immunostaining (magenta). The principal dendrite and axon were indicated by white arrowhead and asterisk, respectively. Scale bar: 20 μm. (B-E) Time-lapse imaging of dendritic development in GFP-transfected pyramidal and granule cells. Cell types were identified by Math2 and Prox1 immunostaining (magenta) after imaging. Asterisks indicate axon; arrows and arrowheads indicate the principal dendrites at the time points of observation. Scale bar: 30 μm. (F) The frequency of replacement of the principal dendrite observed from DIV 4. The replacement of the principal dendrite was counted every 10 hr in 30 min-interval movies. n = 22.

Both pyramidal and granule cells exhibited a polarized shape with a single principal dendrite by DIV 4 (Fig. 5A). However, principal dendrite designation was not fixed; rather, all dendrites were highly dynamic with extensive growth and retraction at this stage. The principal dendrite occasionally receded, and in turn, one of the other minor dendrites rapidly extended to become a new principal dendrite (Fig. 5B and 5C, S3 Movie). Neurons thus maintained a unipolar shape at any point of observation. By DIV 10, the principal dendrite was mostly fixed and rarely replaced by other dendrites (Fig. 5D and 5E). Frequency of replacement of the principal dendrite designation was gradually diminished over the culture period, reaching almost zero by DIV 10 (Fig. 5F). These results suggest that the principal dendrite is determined several days after dendritic polarity is established both in cultured pyramidal and granule cells.

### Localization of the Golgi apparatus in growing dendrites

**Fig 6:**
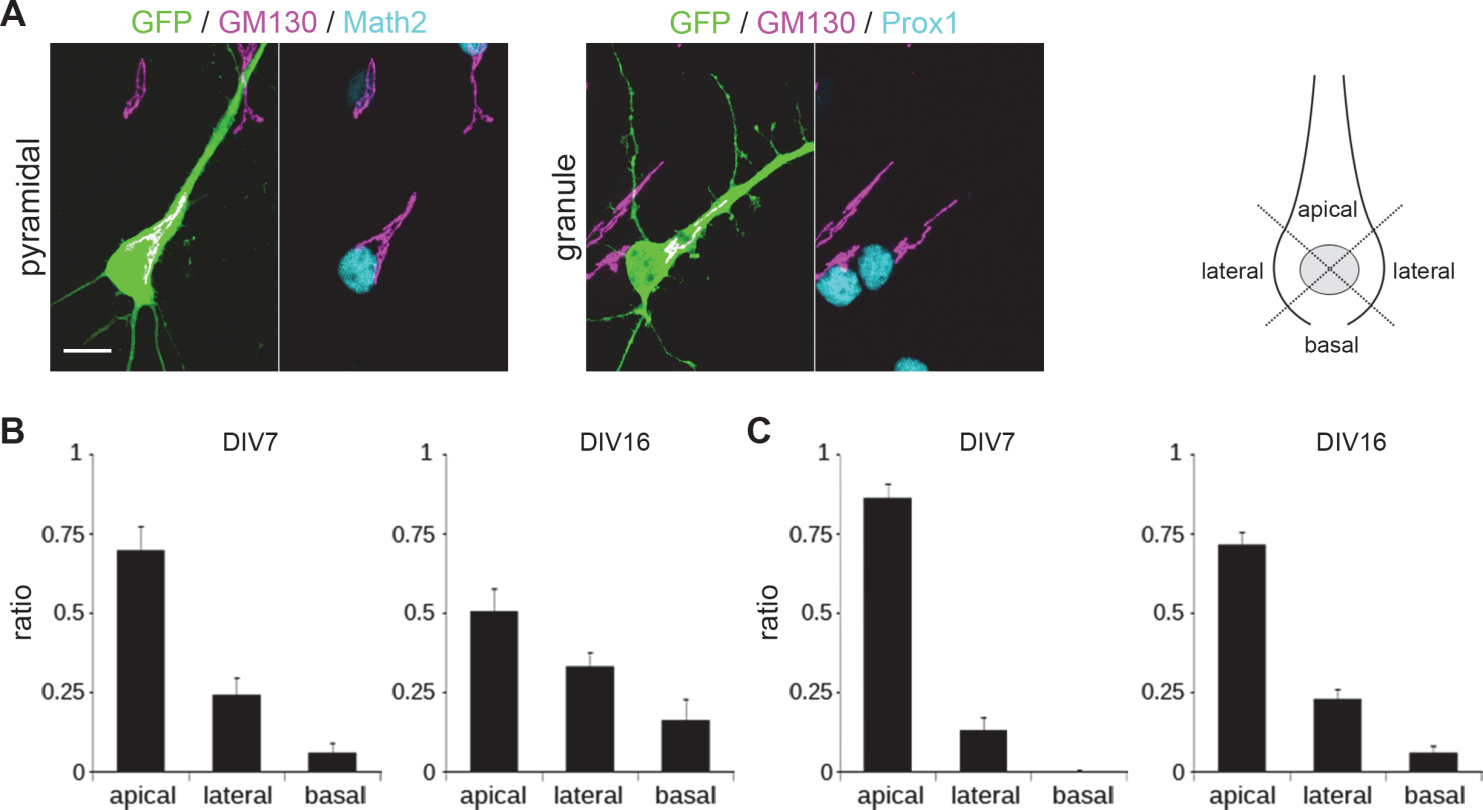
Localization of the Golgi apparatus. (A) Immunofluorescence of the Golgi apparatus with anti-GM130 in pyramidal and granule cells transfected with GFP at DIV 7. Cell morphology and the Golgi were visualized by GFP expression (green) and anti-GM130 immunostaining (magenta). Cell types were identified by Math2 and Prox1 immunostaining (cyan). Scale bar: 10 μm. (B-C) Golgi distribution at DIV 7 and DIV 16 in pyramidal (B) and granule cells (C). Defining the centroid of the nucleus as the origin of a polar coordinate system, the soma was separated into three regions as shown. n = 10.

It has been shown that the Golgi apparatus is polarized toward the principal dendrite in culture and the apical dendrite in pyramidal cells *in vivo* [13]. To confirm whether the Golgi polarizes toward the principal dendrite, we analyzed the distribution of *cis*-Golgi labeled with anti-GM130 antibody in the soma [35] (Fig. 6A). Consistent with previous reports, the somatic Golgi stacks were localized near the base of the principal dendrite in both pyramidal and granule cells (Fig. 6B and 6C). The orientation of the Golgi toward the principle dendrite was more pronounced at DIV 7, when the dendritic polarity was not yet fully established, compared to DIV 16, by which dendritic identities were already stabilized.

**Fig 7:**
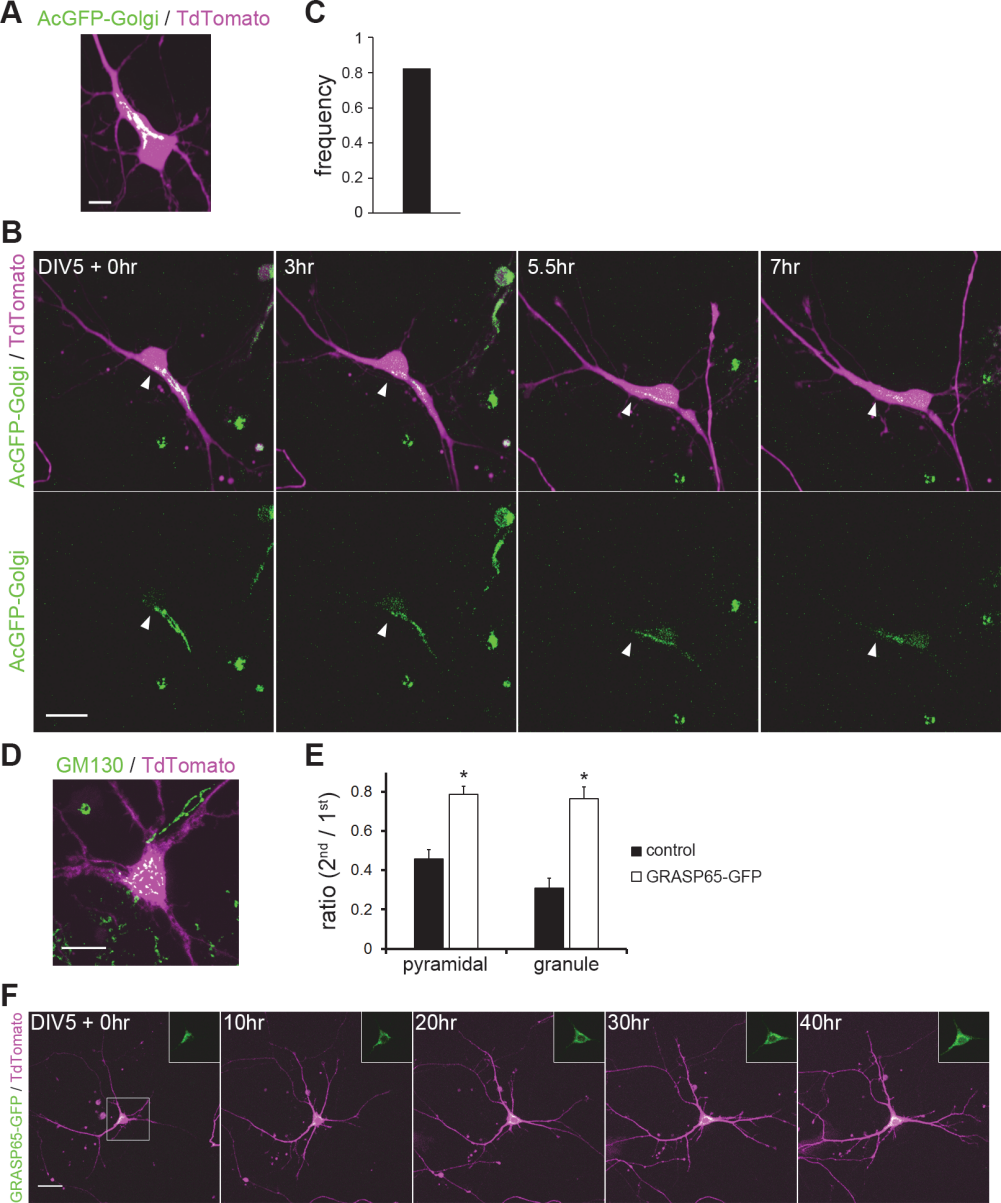
Translocation of the Golgi apparatus. (A) Golgi localization in a neuron transfected with TdTomato (magenta) and AcGFP-Golgi (green). Scale bar: 10 μm. (B) Golgi dynamics during the principal dendrite replacement. Neurons were transfected with TdTomato (magenta) and AcGFP-Golgi (green). White arrowheads indicate the tip of AcGFP-Golgi. Scale bar: 20 μm. (C) The frequency of simultaneous Golgi relocation to the principal dendrite. n = 17. (D) Golgi localization was visualized with anti-GM130 (green) in a neuron overexpressing GRASP65-GFP. Cell was visualized by TdTomato expression (magenta). The Golgi was fragmented and dispersed in the cell soma. (E) Polarity index (ratio of the 2^nd^ to the 1^st^ dendritic length) of control neurons and neurons overexpressing GRASP65-GFP at DIV 10. n = 20 (control), 10 (GRASP65-GFP); Student’s t-test: *P< 0.001. (F) Time-lapse imaging of dendrites and Golgi (inset) in a neuron overexpressing GRASP65-GFP. TdTomato (magenta) and GRASP65-GFP (green) were transfected at DIV 4 and images were taken from DIV 5.

It has been demonstrated that the polarized orientation of the Golgi directs secretory trafficking predominantly toward the apical dendrite for the establishment and maintenance of dendritic polarity in pyramidal cells [13]. This raises a possibility that the Golgi dynamically moves within the soma before the final principal dendrite designation is determined. To test this, we labeled the Golgi by transfecting AcGFP fused with the N-terminal 81 amino acids of human beta-1,4-galactosyltransferase (pAcGFP1-Golgi), and observed the dynamic behavior of the Golgi in live hippocampal neurons at DIV 5. Consistent with the immunofluorescence results using anti-GM130, AcGFP1-Golgi was able to label the Golgi stacks and was often located at the base of the principal dendrite (Fig. 7A). The Golgi actively changed its shape while it remained near the principal dendrite, and it would occasionally invade into the principal dendrite, or expanding to cover a wide area in the soma. When the principal dendrite regressed, the Golgi rapidly relocated to the newly emerged principal dendrite that became the longest among all (Fig. 7B). The frequency of simultaneous Golgi relocation to the principal dendrite was approximately 80% (Fig. 7C). Thus, the establishment of the principal dendrite is preceded by a period in which the Golgi localization was inconstant and transiently polarized toward a tentative principal dendrite among multiple highly dynamic primary dendrites.

We next asked if the change in Golgi localization interfered with the polarization of the principal dendrites. It has been shown that overexpression of the Golgi protein GRASP65 leads to dispersal of the Golgi into multiple dendrites and disruption of apical dendrite specification [13, 36]. Consistent with the previous reports, overexpression of GRASP65-GFP caused vesiculation and dispersal of the Golgi and reduction in dendrite polarization (Fig. 7D and 7E). Time-lapse observation revealed that overexpression of GRASP65-GFP did not decrease the motility of dendrites, but none of them became predominant during DIV 5–7 when control neurons constantly exhibited a polarized shape with a single principal dendrite (Fig. 7F and S4 Movie). These results indicate a critical role of Golgi localization in the principal dendrite specification.

## Discussion

### Morphological differences between pyramidal and granule cells in culture

We identified different cell types included in the conventional hippocampal culture and conducted quantitative assessment of dendritic morphogenesis in pyramidal and granule cells by immunofluorescence and live-cell imaging. Despite slight but significant differences in some morphometric and dynamic properties, overall dendritic morphology of pyramidal and granule cells in culture was remarkably analogous to each other, especially in younger cultures before DIV 13. Unlike *in vivo*, granule cells in culture retained multiple minor dendrites in addition to a single principal dendrite. Although the average size of the somata and dendritic arbors were significantly different, a considerable proportion of cells had an intermediate size, with cell type identification only distinguishable by molecular marker expression.

The primary mouse hippocampal culture utilized in this study has been widely used for analyses of electrophysiological and morphometric properties of pyramidal cells. However, we have shown that granule cells were consistently included in cultures prepared from different aged mice. This is consistent with previous findings that in mice, pyramidal and granule cells emerge simultaneously by E10, while in rats the generation of the two cell types begin at different time points during embryonic development [33, 37]. It was also difficult to completely dissect the Ammon’s horn and dentate gyrus from the tiny embryonic mouse hippocampal formation. Thus, for characterization of pyramidal cells, one should carefully identify neuronal types in primary mouse hippocampal cultures that are likely to include a significant proportion of granule cells.

### Basal dendrites in cultured granule cells

One important structural difference between pyramidal and granule cells is the maintenance of basal dendrites. Pyramidal and granule cells share overlapping gene expression profiles during cell differentiation despite apparent differences in cell morphologies after maturation [38–40]. Recent studies using genetic approaches have shown that Prox1 is a determinant of the dentate granule cell identity over the pyramidal cell fate, and that targeted deletion of Prox1 in postmitotic granule cells induces expression of CA3 pyramidal cell markers and formation of basal dendrites [41]. These studies indicate that the regression of basal dendrites in granule cells requires intrinsic transcriptional regulation. In contrast, the present study revealed that regression of basal dendrites was not complete in culture, implying that pruning of basal dendrites is also dependent on the extracellular environment of the DG. It has been shown that granule cells retain basal dendrites in the epileptic hippocampus [42–45]. Granule cells in dissociated culture may form aberrant excitable inputs that prevent the regression of basal dendrites. Alternatively, continued expression of immature granule cell markers calretinin suggest that signals required for basal dendrite elimination in mature granule cells might not be activated in the primary culture.

### Polarization and specification of apical and basal dendrites

Previous studies using primary rat hippocampal cultures indicated that pyramidal cells in culture do not exhibit dendritic polarity until DIV 8 and final principal dendrite designation was attained by enhanced growth of one of several equivalent dendrites between DIV 8 and DIV 10 [13, 16]. In contrast, primary mouse hippocampal neurons acquired clear dendritic polarity with a single principal dendrite apposed by the Golgi as early as DIV 4. All dendrites and the Golgi were highly dynamic at this stage and the principal dendrite was frequently replaced by one of other minor dendrites. Final principal dendrite designation was established as formation and retraction of primary dendrites gradually ceased around DIV 10.

The apical dendrite in cortical pyramidal cells is thought to originate from the long leading process of unipolar neurons during the long-distance radial migration into the cortical plate [46, 47]. In contrast, hippocampal pyramidal cells and dentate granule cells do not necessarily form a single dominant leading process but frequently display a multipolar shape during the short-distance radial migration from the intermediate zone to the cortical layer of the hippocampal primordium [48, 49]. This suggests that the apical dendrite of hippocampal neurons could arise from one of several equivalent neurites of the multipolar cells instead of from the single leading process of bipolar cells. The determination process of the final principal dendrite designation among multiple neurites might be reflected by the frequent replacement of the principal dendrite in younger cultures.

The replacement of the principal dendrite was accompanied with relocation of the Golgi toward the newly emerged principal dendrite. Furthermore, disruption of polar localization of the Golgi perturbed principal dendrite designation, supporting the idea that the Golgi localization is requisite for the formation of the principal dendrite in hippocampal neurons in culture [13]. Prolonged instability of dendritic polarity and the Golgi localization may be due to the lack of spatial cues in the dissociated culture. Recent studies have indicated that Reelin signaling regulates neuronal polarization and extension of the Golgi into the apical dendrite [14, 50, 51]. We hypothesize that the establishment of dendritic polarity is regulated by a cell-autonomous mechanism, as it was reproduced in dissociated cultures, while environmental cues would guide polarized dendrites into the correct orientation and position. The apical and basal dendrites of pyramidal cells orient oppositely in the hippocampus, suggesting that they are guided by distinct positional cues. The lack of reliable molecular markers for apical and basal dendrites prevented us from confirming whether the minor dendrites differentiated into basal dendrites in culture. Further investigations will elucidate whether the principal dendrite and other minor dendrites differentially respond to guidance cues such as Reelin or semaphorins, and, if so, the molecular mechanisms of spatial distribution of apical and basal dendrites.

## Conclusions

We have characterized the mouse hippocampal cell culture and analyzed the dynamics of dendritic differentiation in pyramidal and granule cells. Cultured granule cells retain basal dendrites and take pyramidal cell-like morphology, suggesting that regression of basal dendrites requires environmental cues in the DG. Polarized growth of a single principal dendrite is evident in early stages, which is frequently replaced by competition among labile dendrites, with final designation determined during differentiation in culture. The present approach provides a useful model system to dissect the molecular mechanisms of apical and basal dendrite formation in pyramidal cells.

## Supporting Information

S1 Fig Cell type identification by immunostaining. Cells were visualized by GFP expression (green), and immunostained with pyramidal cell marker Math2 (cyan) and granule cell marker Prox1 (magenta) at DIV 7. Scale bar: 50 μm.
(TIF)

S1 Movie Time-lapse imaging from DIV 7. Pyramidal and granule cells were visualized by GFP expression.
(MP4)

S2 Movie Time-lapse imaging from DIV 13. Pyramidal and granule cells were visualized by GFP expression.
(MP4)

S3 Movie Time-lapse imaging from DIV 4. Pyramidal and granule cells were visualized by GFP expression.
(MP4)

S4 Movie Time-lapse imaging of a GRASP65-overexpressing neuron. Cell shape was visualized by TdTomato expression. Images were taken from DIV 5.
(MP4)
