# Supplementary figures and images for: Differentiation of Apical and Basal Dendrites in Pyramidal Cells and Granule Cells in Dissociated Hippocampal Cultures
Source: PLoS One. 2015 Feb 23;10(2):e0118482. doi: 10.1371/journal.pone.0118482 (PMC4338060; doi:10.1371/journal.pone.0118482)

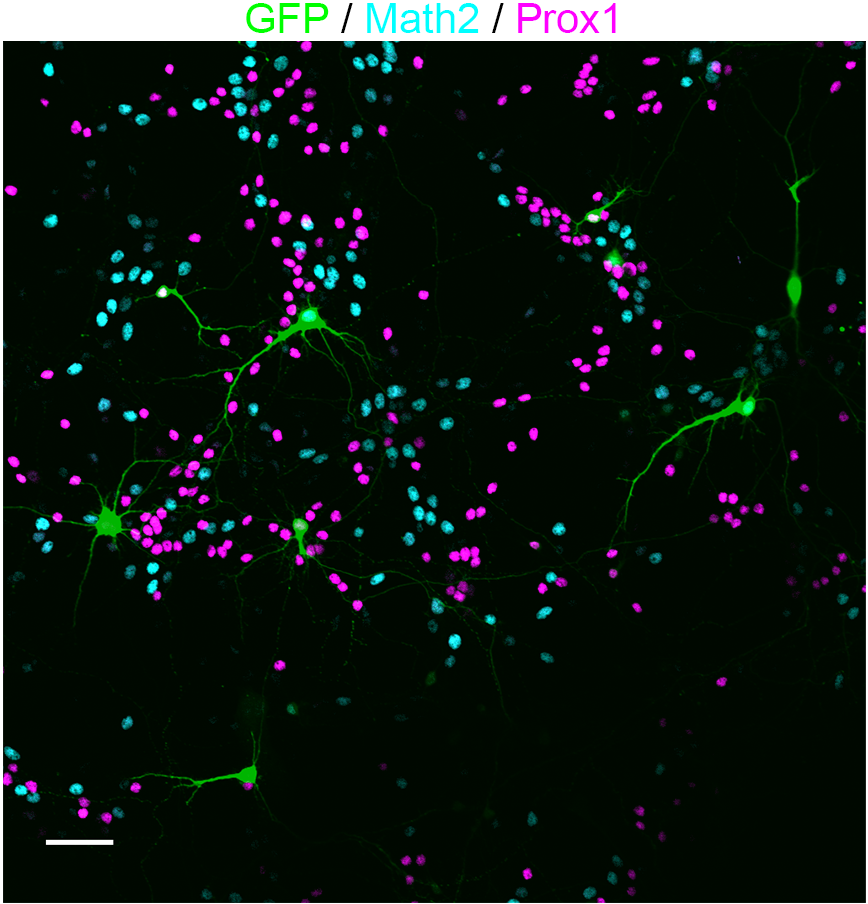

Supplement: S1 Fig — Cells were visualized by GFP expression (green), and immunostained with pyramidal cell marker Math2 (cyan) and granule cell marker Prox1 (magenta) at DIV 7. Scale bar: 50 μm. (TIF) [file pone.0118482.s001.tif]
